# Choice-dependent cross-modal interaction in the medial prefrontal cortex of rats

**DOI:** 10.1186/s13041-021-00732-7

**Published:** 2021-01-15

**Authors:** Mengyao Zheng, Jinghong Xu, Les Keniston, Jing Wu, Song Chang, Liping Yu

**Affiliations:** 1 Key Laboratory of Brain Functional Genomics (Ministry of Education and Shanghai), Key Laboratory of Adolescent Health Assessment and Exercise Intervention of Ministry of Education, and School of Life Sciences, East China Normal University, Shanghai, 200062 China; 2 Department of Physical Therapy, University of Maryland Eastern Shore, Princess Anne, MD 21853 USA

**Keywords:** Behavioral training, Cross-modal interaction, Decision making, Medial prefrontal cortex, Multisensory

## Abstract

Cross-modal interaction (CMI) could significantly influence the perceptional or decision-making process in many circumstances. However, it remains poorly understood what integrative strategies are employed by the brain to deal with different task contexts. To explore it, we examined neural activities of the medial prefrontal cortex (mPFC) of rats performing cue-guided two-alternative forced-choice tasks. In a task requiring rats to discriminate stimuli based on auditory cue, the simultaneous presentation of an uninformative visual cue substantially strengthened mPFC neurons' capability of auditory discrimination mainly through enhancing the response to the preferred cue. Doing this also increased the number of neurons revealing a cue preference. If the task was changed slightly and a visual cue, like the auditory, denoted a specific behavioral direction, mPFC neurons frequently showed a different CMI pattern with an effect of cross-modal enhancement best evoked in information-congruent multisensory trials. In a choice free task, however, the majority of neurons failed to show a cross-modal enhancement effect and cue preference. These results indicate that CMI at the neuronal level is context-dependent in a way that differs from what has been shown in previous studies.

## Introduction

In real life, we often receive multiple sensory cues simultaneously (with most being visual and auditory). The brain must combine them properly and form an effective decision in response to whatever the combination represents accurately. During this process, the brain must decide what sensory inputs are related and what integrative strategy is appropriate. In the past three decades, this process of cross-modal interaction (CMI) or multisensory integration has been widely examined in many brain areas such as superior colliculus, and both primary sensory and association cortices [1–6]. A series of integrative principles that govern this process have been derived (i.e., spatial, temporal, and inverse effectiveness), and testing has shown them to be operant in many brain areas [2]. In the classic example, multisensory neurons in superior colliculus can show greatly enhanced responses to spatiotemporally congruent multisensory cues [7]. Similarly, in monkeys performing a directional task, neurons in several cortical regions such as the dorsal medial superior temporal area have shown enhanced heading selectivity when matched visual and vestibular cues are given simultaneously [8]. In like manner, effectively integrating cross-modal cues was also found to improve perceptual performance [9–11] and shorten reaction times [12–14].

There is an increasing number of studies showing that CMI also could significantly modulate decision-related neural activities in many cortical regions [15]. Psychophysical studies report that perceptual decision-making often relies on CMI [16, 17]. Neuroimaging studies have demonstrated that CMI can directly influence perceptual decisions in both association and sensory cortices [18–20]. Also, in the neuronal level, several studies examined the effect of CMI on perceptual decision-related activities [21–23]. Despite these discoveries, the underlying neural mechanisms of multisensory perceptual decisions remain largely unclear. One of the interesting but challenging questions is what multisensory strategies are employed by the brain to deal with the difference in task contexts.

To explore this, we examined perceptual decision-related activities of the medial prefrontal cortex (mPFC) when rats performed three different cue-guided two-alternative forced-choice tasks. Rodent mPFC receives multimodal cortico-cortical projections from the motor, somatosensory, visual, auditory, gustatory, and limbic cortices [24, 25]. Single neuron activity in mPFC can be considered as a reflection of an ad-hoc mixture of several task-related features such as sensory stimuli, task rules, and possible motor responses [26–28]. Task 1 required rats to discriminate stimuli based on auditory signal alone (two pure tones of different frequencies sometimes paired with an invariant uninformative visual cue) and then make a behavioral choice (left or right). In Task 2, complexity was increased, as the visual cue was made informative for behavioral choice. In Task 3, animals could make a free choice without any cue discrimination. As shown in the following results, these three setups demonstrated that CMI could significantly modulate perceptual decision signals in mPFC in a context-dependent manner.

## Results

**Fig. 1:**
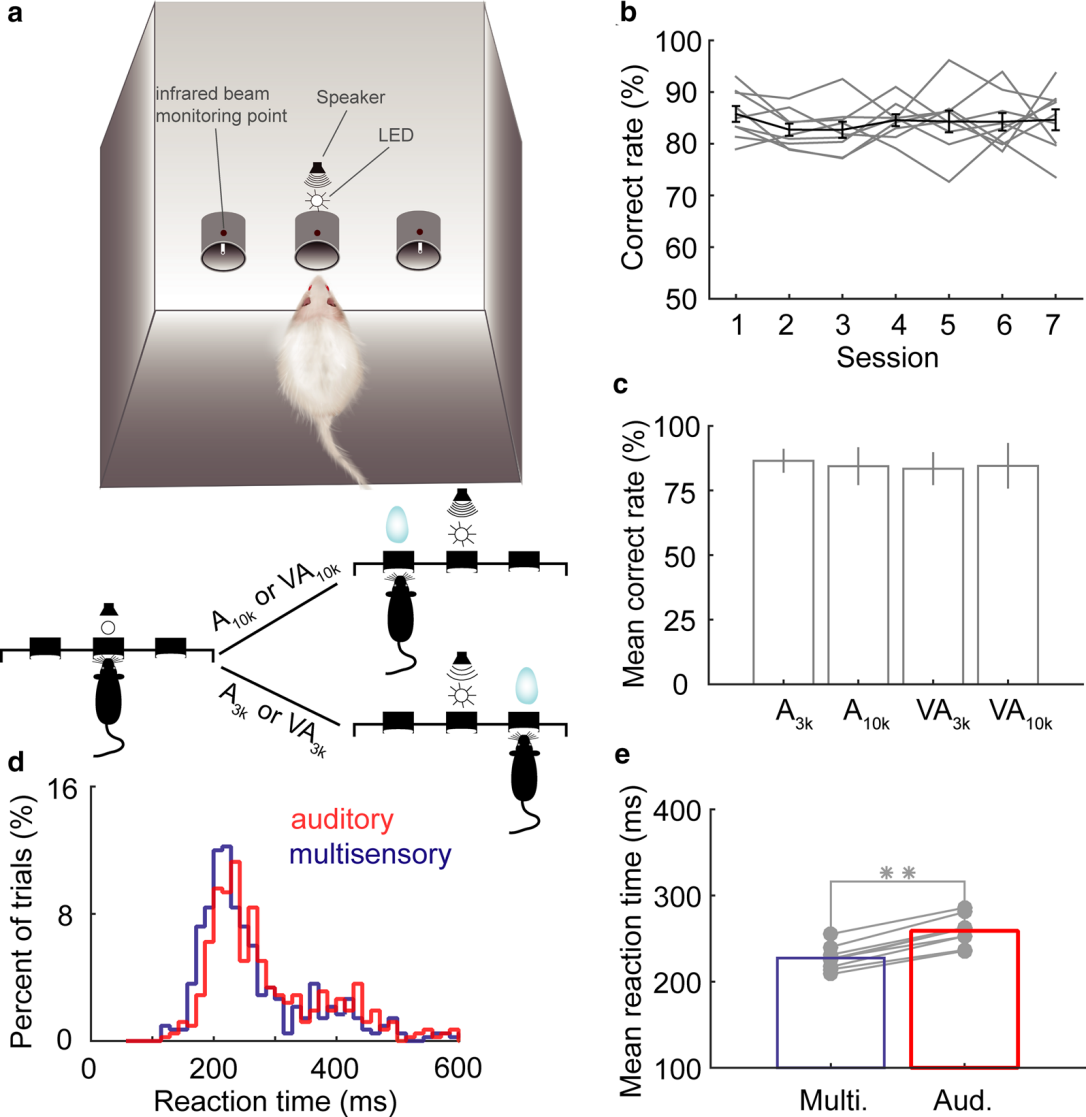
Stimulus discrimination task and behavioral performance. **a** A schematic of the behavioral paradigm used. An individual trial started when trained rats placed their nose in the central port. Next, a lone auditory stimulus or a combined auditory and visual stimulus was presented via a centrally positioned light emitting diode (LED) and speaker to cue the location for a water reward. When the stimulus was either a 10 kHz pure tone (A_10k_) or a combination of a 10 kHz pure tone and a flash of light (VA_10k_), the animal would be rewarded at the left port. If the stimulus given was a 3 kHz pure tone alone (A_3k_) or the same tone paired with a flash of light (VA_3k_), the animal would receive the reward in the right port. Trials of different stimuli combinations (A_3k_, A_10k_, VA_3k_, VA_10k_) were presented in a randomized order. **b** The correct response rate (the number of correct trials divided by the total number of trials, overall mean, black line) are shown for 7 complete testing sessions using 9 well-trained animals (Error bar, SEM). **c** The mean correct rate across all animals for each stimulus condition (Error bar, SEM). **d** The distribution of reaction times for both auditory (gray) and multisensory (black) trials performed by a well-trained animal. **e** A comparison of mean reaction times between auditory and multisensory trials across all animals. **, p < 0.001

We performed three series of experiments. In each experiment, we first trained animals to perform a specific cue-guided two-alternative forced-choice task and then examined mPFC neural activity during the task. All of the behavioral tasks were conducted in a training box (Fig. 1a). In Task 1 (details below), animals were required to make a choice based on whether the auditory stimulus or the auditory component of a multisensory cue, was a lower (3 kHz) or higher frequency (10 kHz) pure tone. Task 2 required animals to discriminate two criteria, the cue modality, and, if multisensory, the frequency content of the auditory component. In Task 3, animals were not required to discern stimuli at all and could make a free choice. These tasks allowed us to investigate how mPFC multisensory perceptual decision strategies changed with the demands of the task.

### The effect of an uninformative visual cue on mPFC neurons' multisensory perceptual decision

A total of 9 rats were trained to perform Task 1 (Fig. 1a). A trial was initiated when a rat poked its nose into the central port in a line of three ports on one wall of the training box (see Fig. 1a). After the waiting period of 500–700 ms, a cue, randomly chosen from a group of 4 cues (3 kHz pure tone, A_3k_; 10 kHz pure tone, A_10k_; 3 k Hz pure tone + flash of light, VA_3k_; 10 kHz pure tone + flash of light, VA_10k_), was presented in front of the central port. Based on the auditory cue, the rat was required to choose a port (left or right) to obtain a water reward within 3 s. If the stimulus was A_10k_ or VA_10k_, the rat should move to the left port for harvesting the water reward (Fig. 1a). Any other cue indicated the animal should move to the right port for a reward. Rats readily learned this cue-guided two-alternative-choice task. After the animals performed the task correctly > 75% of the time in five consecutive sessions, they were deemed well-trained and could then undergo implantation and later electrophysiological recording.

Once well-trained, the average behavioral performance stabilized at 84 ± 2.9% (Fig. 1b). There was no difference in behavioral performance between auditory and multisensory cued trials (Fig. 1c). Despite this, the presence of the visual cue sped up the process of cue discrimination. The reaction time, defined as the temporal gap between the cue onset and the moment when the animal withdrew its nose from the infrared beam monitoring point in the central port (Fig. 1a), was compared between auditory and multisensory trials (Fig. 1d, e). Note that rats responded more quickly in multisensory trials with a mean reaction time of 224 ± 14 ms across animals, significantly shorter than 256 ± 17 ms in auditory trials (t(8) = -15.947, p < 0.00001, paired t-test).

**Fig. 2:**
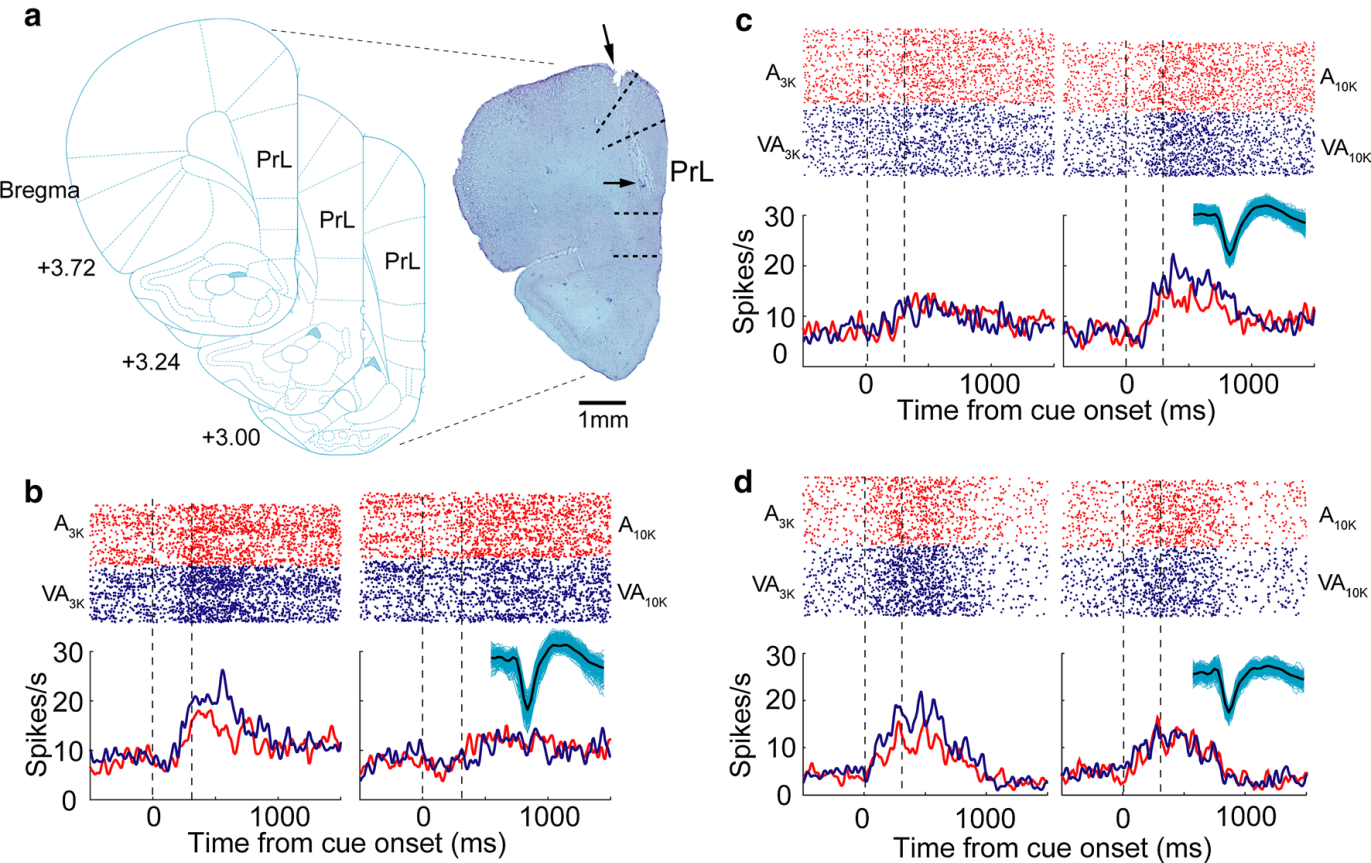
Neuronal activity during task performance and histological verification of recording sites. **a** The photograph of a stained brain section showing the electrode track (top arrow) and the final location of the electrode tip (bottom arrow). All recording sites used were similarly verified to be in the prelimbic area (PrL) of the medial prefrontal cortex. In **b** rasters (top rows) and peri-stimulus time histograms (PSTHs, bottom traces) showed a neuron’s activities in A_3k_ (left, red), VA_3k_ (left, blue), A_10k_ (right, red), and VA_10k_ (right, blue) trials. Inserted is action potentials of this example mPFC neuron (2000 single waveforms and their average, black). Mean spike counts of correct trials were computed in 10-ms time windows and smoothed with a Gaussian (σ = 100 ms). Responses were aligned to the initial cue presentation. In multisensory trials, visual and auditory stimuli were presented simultaneously. Dashed lines denote the stimulus onset and offset. In the same way, **c**, **d** show two more example neurons

**Fig. 3:**
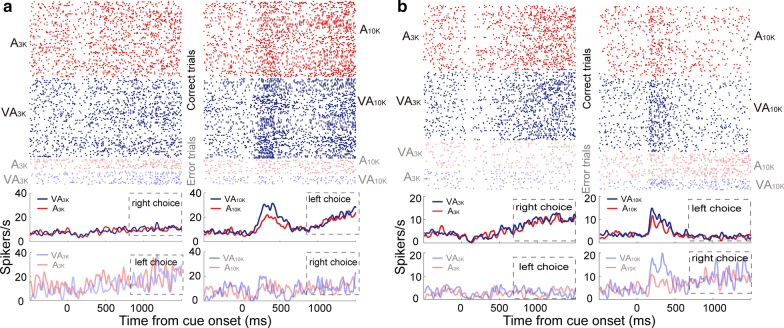
Cue-categorization and behavioral-choice related activity in mPFC neurons. **a** Rasters and PSTHs showed a neuron’s activities in both correct (dark color) and error (light color) trials of each given cue condition. Note that the cue-categorization signal preceded a behavioral choice signal denoted by a dashed rectangle. **b** Another example neuron. The conventions used are the same as in Fig. 2

We used tetrode recordings to characterize the task-related activity of individual neurons in left mPFC while well-trained rats performed Task 1 (Fig. 2a). On average, animals performed 266 ± 53 trials in a daily session. A total of 654 neurons were recorded (65 ± 14 neurons per animal), and their responses were examined. 313 of them appeared to show cue-categorization signals within 500 ms after the cue onset (firing rate in continuous three bins >  = spontaneous firing rate, Mann–Whitney Rank Sum Test, *p* < 0.05), and all further analysis was focused on them. In the examples shown in Fig. 2b-d, cue-categorization signals appeared to discriminate well auditory pure tones (low vs. high) and sensory modalities (multisensory vs. auditory). For instance, as is shown in Fig. 2b, the response in A_3k_ trials is higher than in A_10k_ trials, and the firing rate in VA_3k_ trials is higher than in A_3k_ trials. Nearly 34% (107/313) of neurons examined showed both cue-categorization signals and behavioral choice signals (coding moving directions). These two signals could be easily separated because behavioral choice signals occurred much later than cue-categorization signals (typically later than 600 ms after cue onset) (Fig. 3a, b). Different from cue-categorization signals, behavioral choice signals usually showed no difference between multisensory and auditory trials (Fig. 3a, b).

**Fig. 4:**
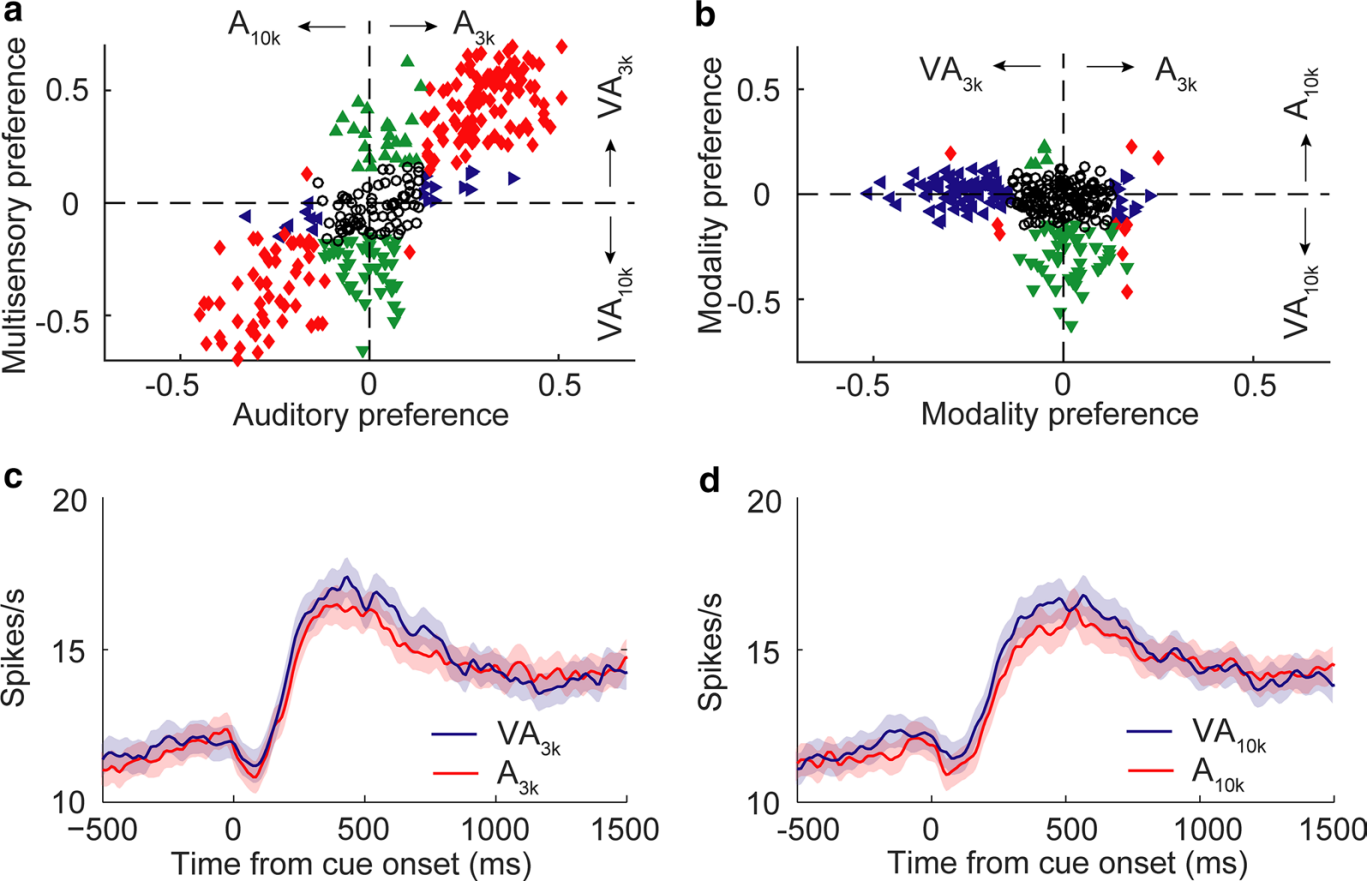
Distributions of neuronal choice preferences and mean responses. **a** Choice preferences (ROC value) for both auditory and multisensory responses are shown. Each symbol shows the value of a single neuron. Abscissa, auditory choice preference for A_10k_ vs. A_3k_ trials; ordinate, multisensory choice preference for VA_10k_ vs. VA_3k_ trials. Open circles: neither auditory nor multisensory choice preference was significant (p < 0.05, permutation test, 5000 iterations); triangles: either multisensory (green) or auditory (blue) choice preference was significant; red diamonds: both multisensory and auditory choice preferences were significant. Similarly, in **b** modality choice preference (auditory vs. multisensory) are shown. Dashed lines represent zero ROC values. **c**, **d** PSTHs show mean responses across populations for different stimulus trials. Shaded areas, SEM

We used ROC analysis to generate an index of auditory choice preference that measures how strongly a neuron's cue-categorization signal for A_3k_ trials diverged from the cue-categorization signal for A_10k_ trials. In the same way, an index of multisensory choice preference was defined. As shown in exemplar cases (Figs. 2b, c, 3a, b), nearly half of neurons examined (55%, 171/313; preferring A_3k_: N = 111; preferring A_10k_: N = 60) exhibited an auditory choice preference (permutation test, *p* < 0.05). However, more neurons (71%; 222/313) showed the multisensory choice preference (Fig. 4a), in that, a sizeable minority of neurons (23%, 72/313) showed the perceptual choice preference only between two multisensory conditions (see the example in Fig. 2d). This result indicated that the visual cue, albeit uninformative, was able to facilitate mPFC neurons’ auditory choice capability. Auditory and multisensory choice preferences were fairly consistent. In other words, if the neuron preferred A_10k_ it usually preferred VA_10k_ (Fig. 4a).

Taking things further, we examined the influence of visual cue on auditory choice signals. We found that in 49% (155/313) of cases, the simultaneous presentation of a visual stimulus could significantly modulate the response in one or both auditory conditions (permutation test, *p* < 0.05, Fig. 4b). Cross-modal enhancement was the favored processing strategy in use here because, for most neurons (87%, 135/155), the response in VA_3k_ or/and VA_10k_ trials were significantly higher than that in corresponding auditory trials (A_3k_: 79%, 76/96; A_10k_: 88%, 61/69). Due to this, across the population (n = 313), the mean response in multisensory trials was a bit larger than that in corresponding auditory trials regardless of the auditory component frequency (Fig. 4c, d).

**Fig. 5:**
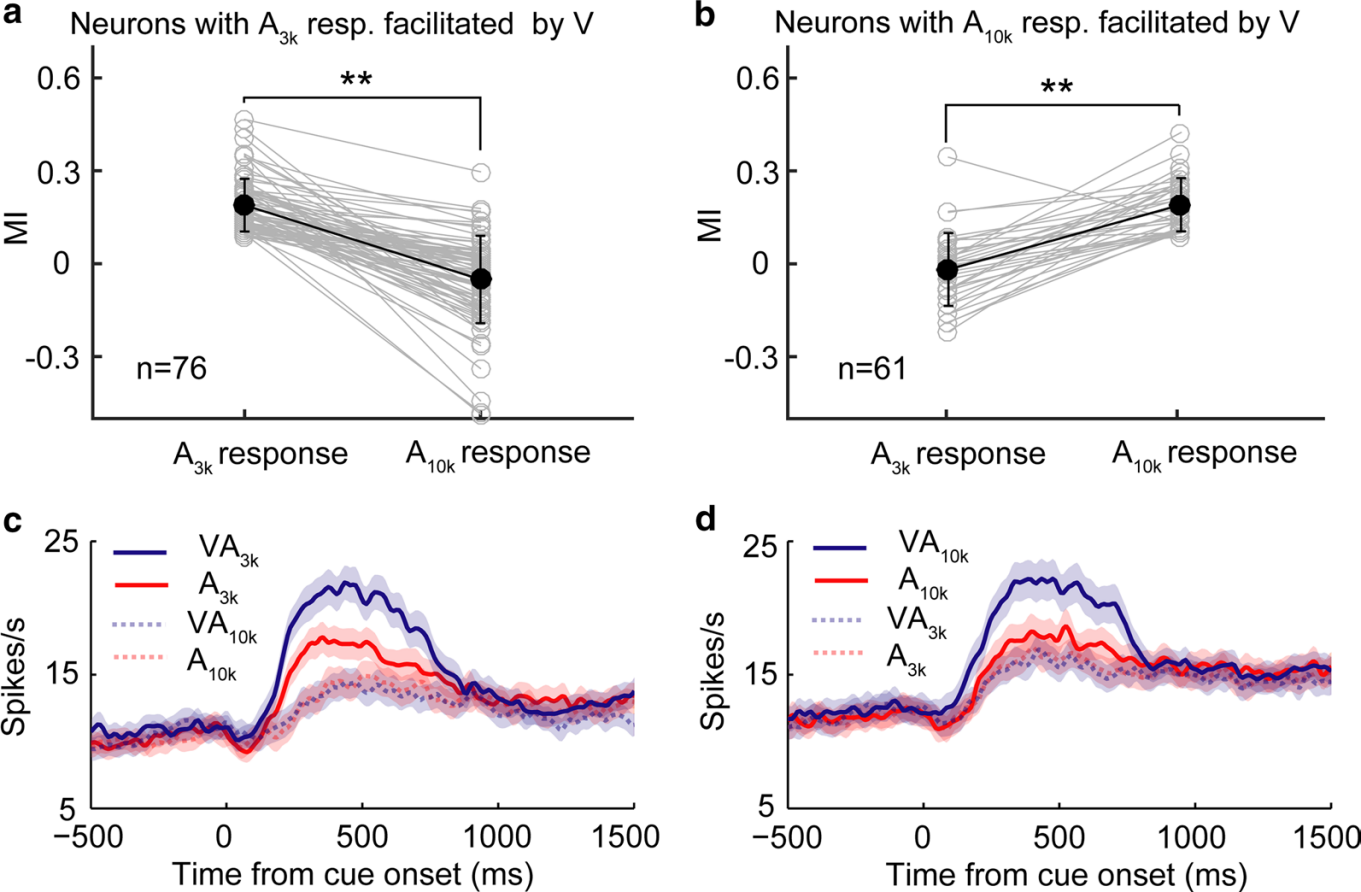
mPFC neurons exhibit a differential pattern of cross-modal interaction. **a** The comparison of the index of cross-modal interaction (MI) between low (3 kHz) and high (10 kHz) tone conditions for neurons with showing cross-modal facilitation in VA_3k_ condition. MI is calculated by the following function: MI = (R_VA_—R_A_) / (R_VA_ + R_A_); where R_VA_ and R_A_ represent the mean response in multisensory and auditory alone trials, respectively. Paired gray circles connected with a gray line represent one neuron’s responses. Dark circles represent the mean MI across neurons. **, p < 0.001. Similarly, **b **shows MI comparisons for neurons with showing cross-modal facilitation in VA_10k_ condition. **c**, **d** shows the mean PSTHs of different cue trials for the same two groups of neurons shown in **a** and **b**, demonstrating greater responsiveness in the multisensory stimulation containing the preferred auditory stimulus

To further investigate those neurons with auditory choice signal facilitated by a visual cue, we were surprised to find that in nearly all of cases (98%, 133/135), the addition of a visual stimulus only facilitated the response in one auditory condition (p < 0.05, permutation test, Fig. 5a, b). We used MI to quantify the effect of cross-modal interaction. In 76 neurons showing cross-modal enhancement in VA_3k_ trials, the mean MI in the VA_3k_ condition was 0.18 ± 0.09, but the mean MI in VA_10k_ condition was near zero (-0.05 ± 0.14, p < 0.0001, Wilcoxon Signed Rank Test, see Fig. 5a). It was also the case in those showing cross-modal enhancement in VA_10k_ trials (n = 61, mean MI: 0.20 ± 0.11 in VA_10k_ condition vs. -0.03 ± 0.12 in VA_3k_ condition, p < 0.0001, Wilcoxon Signed Rank Test, Fig. 5b). Furthermore, we found that the visual cue usually just enhanced the preferred auditory choice signal (see examples in Fig. 2b, c) regardless of whether the preferred was A_3k_ (51/54) or A_10k_ (27/33). Such biased enhancement further strengthened neurons’ choice selectivity (Fig. 5c, d).

### The influence of information congruence/incongruence between visual and auditory cues on mPFC neurons' cross-modal interaction

**Fig. 6:**
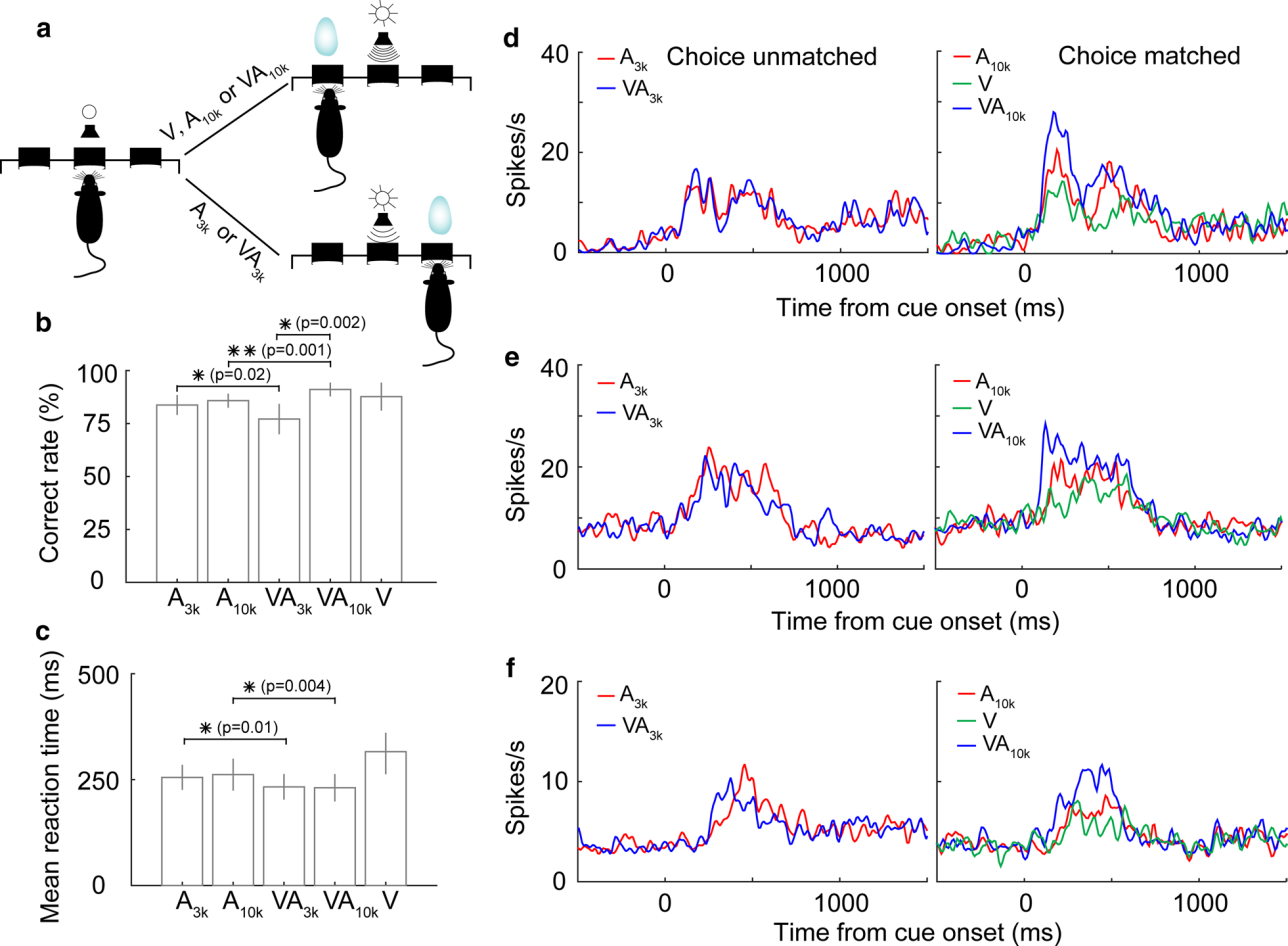
Behavioral performance and neural responses when animals performed Task 2. **a** Schematic of the behavioral paradigm. When the triggered cue is A_10k_, V, or VA_10k_, the animal should move to the left port to obtain a water reward. Any other combination indicates they should go to the right port for the reward. **b** The behavioral accuracy for different cue trials across all animals. **c** The mean behavioral reaction time to each cue combination used across all animals. **d**–**f** PSTHs show the mean response to different cue trials for three neurons

In behavioral Task 1, animals made their behavioral choice based on the auditory cue alone. We then wondered how mPFC neurons would change their integrative strategy if the behavioral choice became dependent on both auditory and visual cues. To examine this, we trained 7 rats to perform a new behavioral task (Task 2). In this task, the only difference from Task 1 is that an individual visual stimulus (V) was introduced into the stimulus pool as an informative cue. If the triggered stimulus is A_10K,_ VA_10k_, or V, animals should go to the left port to get the reward (Fig. 6a). Otherwise, they should move to the right port to be rewarded. This task took animals about two months of training to surpass 75% correct performance for five consecutive sessions. Although there was no difference in behavioral performance between two auditory alone conditions (A_3k_ vs. A_10k_: 83.7% vs. 85.7%, t(6) = 0.888, *p* = 0.41, paired t-test, Fig. 6b), the task showed a difference between two multisensory conditions. This performance increased when the cues themselves had congruent information content and declined when they indicated a cued directional mismatch (VA_3k_ vs. VA_10k_: 77.1% vs. 91.1%, t(6) = 5.214, *p* = 0.002, paired t-test, Fig. 6b). The mean reaction time in multisensory trials across animals was still significantly shorter than that in corresponding auditory trials regardless of whether the auditory is A_3k_ or A_10k_ (A_10k_ vs. VA_10k_: 263 ± 92 ms vs. 232 ± 79 ms, t(6) = 4.585, *p* = 0.004, paired t-test; A_3k_ vs. VA_3k_: 256 ± 73 ms vs. 234 ± 75 ms, t(6) = 3.614, *p* = 0.01, paired t-test; Fig. 6c). There was no difference in the reaction times between two multisensory conditions (t(6) = 0.0512, *p* = 0.961, paired t-test).

**Fig. 7:**
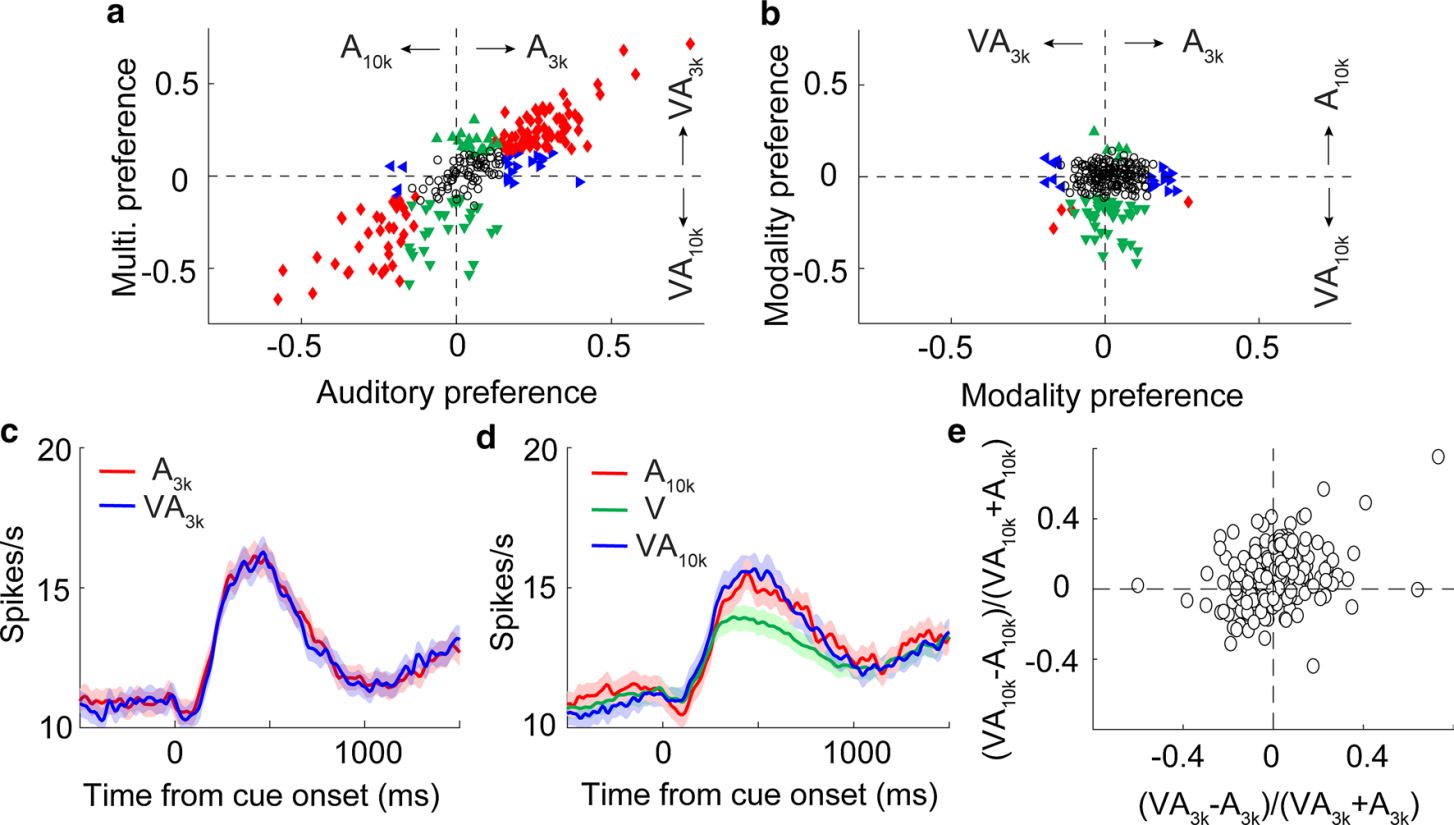
Cue preferences, neural responses, and multisensory integration. **a** Neuron response preference by modality (auditory vs. multisensory). **b** Preference for auditory response (x-axis) against multisensory response (y-axis). **c**, **d** The mean PSTHs of different cued trials across neurons. **e** The comparison of MIs between the two different auditory conditions for all neurons tested (3 kHz vs. 10 kHz). The conventions used are the same as in Fig. 4

We examined the responses of 456 mPFC neurons recorded during performing Task 2. 54% (247/456) of these neurons showing cue-categorization signals (see examples in Fig. 6d-f). The result showed that the introduction of an informative visual stimulus into the cue pool significantly affected mPFC neurons’ CMI strategy (Fig. 7a, b), one which was dependent on information content. Compared with Task 1, a far lower proportion of neurons (10%, 24/247 in Task 2; 24%, 76/313 in Task 1; X^2^ = 19.96; *p* < 0.00001) showed cross-modal enhancement in VA_3k_ trials (Fig. 7b). indicating that information mismatch disrupted cross-modal enhancement. However, this proportion In information-congruent VA_10k_ trials is similar to the observation in Task 1 (22%, 55/247 in Task 2; 19%, 61/313 in Task 1; X^2^ = 0.65; *p* = 0.42). As shown in Fig. 6d-f, in each case, only the response in VA_10k_ condition was significantly enhanced. Mean responses across the populations tested (n = 247) are shown in Fig. 7c, d. Of these neurons (n = 55) showing cross-modal enhancement in the information-congruent VA_10k_ trials, 20 of them (36%) favored A_10k_ (see the example in Fig. 6d) and 28 of them (51%) showed no overt preference of auditory choice (see the example in Fig. 6e). In several cases, like the neuron shown in Fig. 6f, the visual stimulus appeared to reverse selectivity, and for auditory, they showed a preference for A_3k_, but for multisensory, favored VA_10k_.

The mean MI in information-incongruent VA_3k_ condition across populations (n = 247) is nearly zero (0.01 ± 0.16), which was significantly lower than 0.07 ± 0.15 in the congruent VA_10k_ condition (*p* < 0.00001, Mann–Whitney Rank Sum Test; see the comparison of an individual case in Fig. 7e). Also, one would expect that the information match should induce more substantial effects of cross-modal enhancement. It was not the case, however. In examining all neurons exhibiting cross-modal enhancement in VA_10k_ condition in Task 1 and Task 2, we found no difference between them (mean MI: 0.21 ± 0.18 in Task 2 vs. 0.20 ± 0.11 in Task 1, *p* = 0.489, Mann–Whitney Rank Sum Test). Summarily, these results indicate that the activities of mPFC neurons reflected the context of the task and maintained their ability to discriminate, and, ostensibly, aid in successful task completion.

### Cross-modal interaction in a choice-free task

**Fig. 8:**
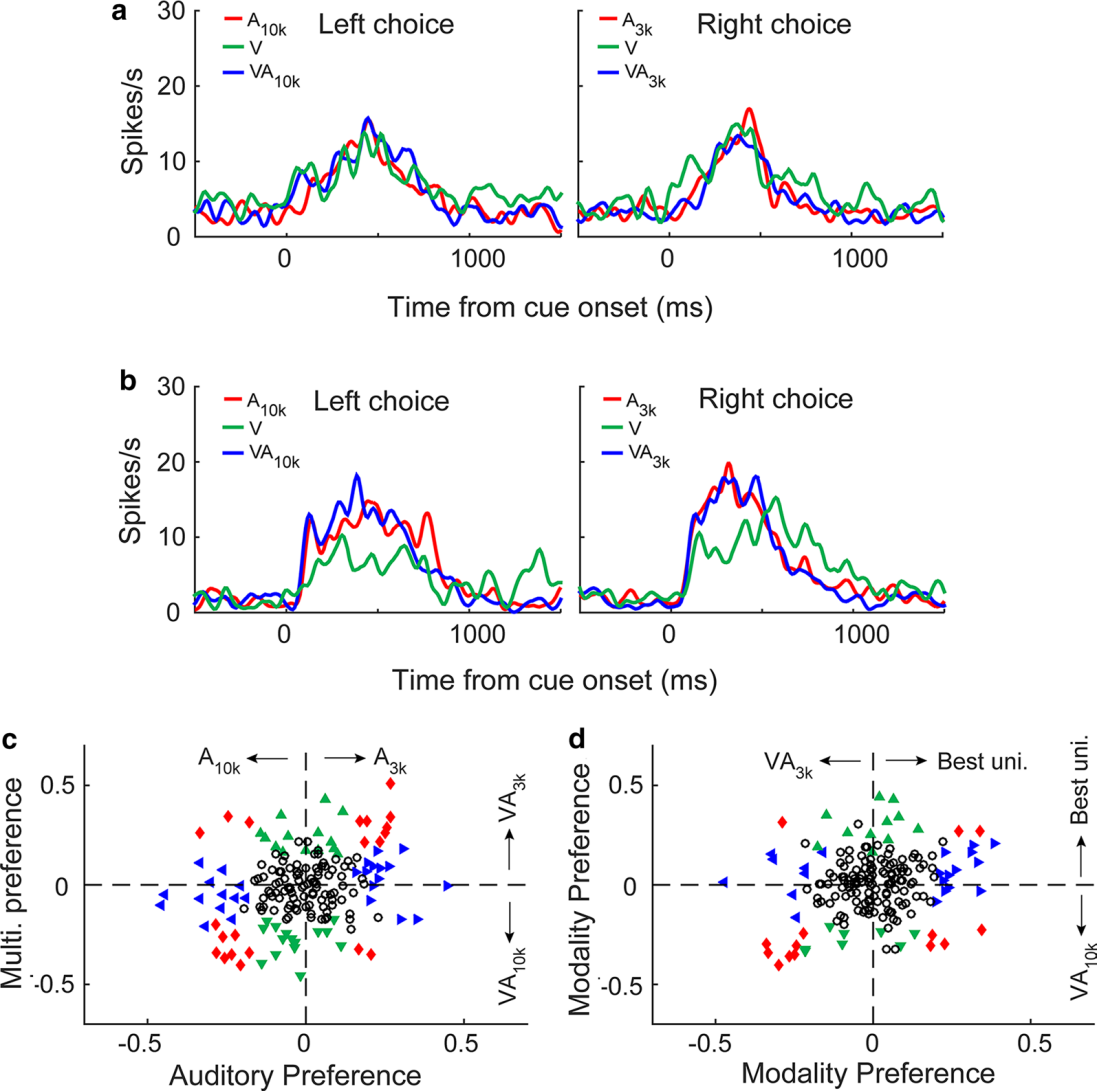
mPFC neurons’ activities and choice preferences in a choice-free behavioral task. **a**, **b**, PSTHs show the mean response to different cue trials for two neurons. **c** Auditory vs. multisensory choice preferences is shown. **d** Neurons’ preferences for the modality (unisensory vs. multisensory). Conventions are the same as in Fig. 2 and in Fig. 4

Tasks 1 and 2 required animals to discriminate sensory cues. The next intriguing question to us was how then mPFC neurons would treat different combinations of sensory cues and CMI when cue discrimination is not required? To investigate this, we trained another group of rats (n = 9) to perform a choice-free task (Task 3). In this task, animals would get a water reward in either the left or right port regardless of which stimulus was presented, rendering the cueing discrimination irrelevant. We carefully examined 184 mPFC neurons recorded during the performance of Task 3. For consistency with the earlier analyses, neuron’s response in A_3k__right_choice trials was compared with the response in A_10k__left_choice trials, and so was done in multisensory comparison. Different from those recorded in Task 1&2, in Task 3, the majority of mPFC neurons examined failed to show auditory choice preferences (74%, 137/184) and correspondingly, multisensory choice preference (73%, 135/184). Figure 8a shows such an example. Population distributions for choice selectivity are illustrated in Fig. 8c. This was also the case in the comparison of responses between conditions of the same moving direction (right direction: auditory choice selectivity, 75%, 138/184; multisensory choice selectivity, 72%, 132/184; left direction: auditory choice selectivity, 77%, 142/184; multisensory choice selectivity, 71%, 130/184).

For the majority of neurons (72%, 132/184), their response in multisensory trials is very similar to the corresponding response in auditory or visual trials (p > 0.05, permutation test, see the example in Fig. 8a, b and populations in Fig. 8d). For those neurons with the response in auditory trials that was influenced by visual stimulus (28%, n = 52), they showed induced inhibitory or facilitatory effects that appear similar (facilitated: 24; inhibited: 23; facilitated and inhibited: 5, see Fig. 8d).

We carefully examined neurons exhibiting auditory choice selectivity (n = 49) to see whether visual cue, as what we observed in cue-discrimination tasks, could specifically induce facilitative effect to the preferred response. The vast majority of cases (44/49) failed to do so, however. Figure 8b showed such an example where the neuron favored the A_3k_ over A_10k,_ but neither response was heightened by the visual stimulus. The mean MIs for both conditions were similar (preferred vs. non-preferred: − 0.03 ± 0.27 vs. − 0.04 ± 0.27; p = 0.441, Mann–Whitney Rank Sum Test). This result, taken together with those given above, reveals that the differential neural activities in mPFC likely reflect the context of the given task. When stimulus discrimination is not required, the neuronal activity exhibits no selectivity. When demanded by an appropriate task, mPFC neurons are quite capable of sensory discrimination.

## Discussion

We used cue discrimination tasks to understand context-dependent CMI in rat mPFC, an area that is believed to be essential both for perception and decision-making. The result showed that, in a task requiring auditory discrimination, the presence of an uninformative visual stimulus mostly served only to heighten the preferred auditory choice signal. As a result, the neurons exhibited better perceptual decision capability for multisensory conditions than for auditory alone conditions. However, if a visual cue, like the auditory, was made informative, mPFC neurons frequently showed a different CMI pattern with an enhanced multisensory perceptual signal when both auditory and visual cues indicated the same behavioral instruction. When no cue discrimination was required in the task, the majority of neurons failed to show the same pattern of CMI and a similar choice strategy. This result greatly expands our understanding of the role that CMI can play in the brain.

Most of our understandings regarding CMI were developed using anesthetized or passively sensing animals. In these studies, the spatiotemporal arrangement and intensities of stimuli were found to be critical for CMI. We believe that more factors should influence CMI when humans and animals perform tasks. Also, as we know now, the levels of neural activity found in an alert, active brain are dramatically different from an anesthetized or passive preparation. To date, few studies have examined the association of multisensory cues (especially visual and auditory) in awake, unrestrained animals [29–31]. Based on our limited knowledge, no study examined the diversity of CMI at the neuronal level during tasks. Thus, our present study provides essential evidence for fully understanding CMI in the brain.

Our result demonstrated that task contexts significantly influenced the strategy of multisensory perceptual decisions. As behavioral demands of a complex decision rose, so could multisensory decision-making strategies. This result is consistent with most of the previous studies that contended contextual representations influenced the way stimuli, events, or actions were both encoded and interpreted [32–34]. These observations were considered especially true in higher-order cortices [25, 35]. Also, in rodents, mPFC has been identified as critical for changing strategies [36–38]. Thus, our result provided new evidence for backing this conclusion. However, it remains to be discovered whether this context-dependent CMI is unique to mPFC or if it exists in other brain areas, which we intend to examine in future studies. Also, the brain state should be a critical factor for influencing CMI, and a recent study showed that cross-modal inhibition dominated in mPFC in anesthetized rats [39].

When performing the task, rats showed shorter reaction times in multisensory conditions. This result is consistent with what was observed in many previous studies conducted in both humans and animals [40–43]. We believe that visual signals, even if task-irrelative, could be integrated into the auditory processing in task engagement, which should help explain why visual cues could speed up auditory discrimination. Previous studies showed that visual inputs could significantly influence the auditory response even in the auditory cortex [44]. Similar studies showed that task-irrelevant sounds could activate human visual cortex [45] and improve visual perceptual processing [10]. Also, multisensory cues could activate more attention effect [46], which might also help facilitate perceptual discrimination. In addition, the effect of cross-modal modulation on mPFC response was not closely correlated with the reaction time. In Task 2, there is no difference in reaction time between congruent and incongruent conditions; whereas, mean MI in the congruent condition was significantly larger than that in the incongruent condition. Of especial relevance to our finding here, it has been shown that a task-irrelevant auditory stimulus could shorten the reaction time of responding to visual cues [47] and multisensory processing of both semantic congruent and incongruent stimuli could speed up reaction time [42]. Our testing in Task 1 failed to show a higher degree of accuracy in choice selection for multisensory conditions. In considering this result, we attribute this to two factors: the completely uninformative character of the visual stimulus as a cue (lacking in *any* information, such as location or direction) and the possible presence of a ceiling effect on performance in a minimalist task with only two choices.

In Task 2, the rats exhibited a higher rate of behavioral performance in the information-congruent multisensory condition. This result is consistent with the multisensory correlation model that describes that multisensory enhancement increases with the rising correlation between multisensory signals [48]. Also, evidence from both human and nonhuman primates showed that information congruence is critical for multisensory integration [49–51]. For example, congruent audiovisual speech enhances our ability to comprehend a speaker, even in noise-free conditions [52], and semantically congruent multisensory stimuli result in enhanced behavioral performance [53]. Conversely, when incongruent auditory and visual information is presented concurrently, it can hinder a listener's perception and even cause him or her to perceive information that was not presented in either modality [54]. In Task 2, A_3k_ and visual cue indicated the different behavioral choices. However, in multisensory VA_3k_ conditions, rats mostly made their choice based on the auditory rather than visual components. This result is consistent with a recent study showing auditory dominance over vision during the integration of audiovisual conflicts [55].

Rat mPFC can be separated into multiple different subregions including the medial agranular cortex, the anterior cingulate cortex, the prelimbic (PrL), and infralimbic (IL) cortices, based on efferent and afferent patterns of projection [56, 57]. Functionally, PrL, the area that we examined in this study, is implicated in perception-based decision making and memory [36, 58–60] and also tuned to the value of spatial navigation goals [61, 62]. Perceptual decision-making is a complex neural process, including the encoding of sensory information, the calculation of decision variables, the application of decision rules, and the production of motor response [63]. In this study, we failed to know whether CMI occurred before or during the process of perceptual decision exactly. There is substantial physiological and anatomical evidence for cross-modal interactions in primary and non-primary sensory cortices [10, 44, 64, 65]. Considering mPFC receives a vast array of information from sensory cortices [56, 66], the effect of CMI might first occur in sensory processing and then influenced the process of decision making in mPFC.

Cross-modal enhancement appears not to be the default integrative mode for mPFC neurons of awake rats because most of them failed to show it in the choice-free task (Task 3). Similar results were found in other studies [67–69]. However, this result is quite different from earlier studies conducted in the superior colliculus and other sensory cortical areas that primarily showed enhanced multisensory responses to spatiotemporally congruent cues [3, 4, 70, 71]. This is reasonable, considering that different brain areas have different functional goals. For instance, it is well understood that the intrinsic functions of superior colliculus include the localization of novel stimuli and cue-triggered orientation. In contrast, the prefrontal cortex is known to be involved in higher-order cognitive functions, including decision making. It is, therefore, reasonable to conclude that different brain regions would need to apply different strategies of CMI to process multisensory inputs in line with their overall processing goals.

## Methods

### Rat subjects

Animal procedures were approved by the Local Ethical Review Committee of East China Normal University and carried out in accordance with the Guide for the Care and Use of Laboratory Animals of East China Normal University. Twenty-five adult male Sprague Dawley rats, provided by the Shanghai Laboratory Animal Center (Shanghai, China) were used for the experiments. These animals were 250–300 g each and were 4–6 months old at the start of behavioral training. Each was housed as one animal per cage under constant temperature (23 ± 1 °C) with a normal diurnal light cycle. All animals had access to food ad libitum at all times. Water was restricted only on experimental days up to the behavioral session and was unrestricted afterward for 5 min. Animals usually trained 5 days per week, in one 50 to 80-min session per day, held at approximately the same time of day. Bodyweight was carefully monitored and kept above 80% of the age-matched control animals undergoing no behavioral training.

### Behavioral task

The animals were required to perform a cue-guided two-alternative forced-choice task slightly modified from other published protocols [72, 73]. Automated training was controlled using a custom-built program running on Matlab 2015b (Mathworks, Natick, Ma. USA). The training was conducted in an open-topped custom-built operant chamber made of opaque plastic (size: 50 × 30 × 40 cm, length × width × height) inside a well-ventilated painted wooden box covered with convoluted polyurethane foam for sound attenuation (outer size: 120 × 100 × 120 cm). Three snout ports, each monitored by a photoelectric switch, are located on one sidewall of the operant chamber (see Fig. 1a). The signals from the photoelectric switches were first fed to an analog–digital multifunction card and digitized (DAQ NI 6363, National Instruments, Austin, TX, USA) and sent via USB to a PC running the training program.

Rats initiated a trial by poking their nose into the center port. Following a short variable delay (500-700 ms), a stimulus (two auditory, two auditory-visual, or one visual, randomly selected) was presented. After presentation of this cue, rats could immediately initiate their behavioral choice, moving to the left or right port (Fig. 1a). If rats made a correct choice (hit trial), they could obtain a water reward, and a new trial could immediately follow. If animals made wrong or no behavioral choice within 3 s after cue onset, the punishment of a 5–6 s timeout was applied.

The auditory cue was delivered via a speaker (FS Audio, Zhejiang, China), using a 300 ms-long 3 kHz (low) or 10 kHz (high) pure tone with 25 ms attack/decay ramps given at 60 dB sound pressure level (SPL) against an ambient background of 35–45 dB SPL. SPLs were measured at the position of the central port (the starting position). The visual cue was a 300 ms-long flash of white light given at 5 ~ 7 cd/m^2^ intensity, delivered by a light-emitting diode. The auditory-visual cue (multisensory cue) was the simultaneous presentation of both auditory and visual cues.

### Assembly of tetrodes

Formvar-Insulated Nichrome Wire (bare diameter: 17.78 μm, A-M systems, WA, USA) was twisted in groups of four as tetrodes (impedance: 0.5–0.8 MΩ at 1 kHz). Two 20 cm-long wires were folded in half over a horizontal bar for twisting. The ends were clamped together and manually twisted clockwise. Finally, their insulation coating was fused with a heat gun at the desired level of twist and cut in the middle to produce two tetrodes. To reinforce each tetrode longitudinally, each tetrode was then inserted into Polymide tubing (inner diameter: 0.045 inches; wall: 0.005 inches; A-M systems, WA, USA) and fixed in place by cyanoacrylate glue. An array of 2 × 4 tetrodes were then assembled using an inter-tetrode gap of 0.4–0.5 mm. After assembly, the insulation coating of each wire was gently removed at the tip, and then the wire was soldered to a connector pin. The reference electrode used was a tip-exposed Ni-Chrome wire of diameter 50.8 μm (A-M systems, WA, USA), and a ground electrode was a piece of copper wire of the diameter of 0.1 mm. Both of these were also soldered to a connector pin. The tetrodes and reference were then carefully cemented by silicon gel and trimmed to an appropriate length immediately before implantation.

### Electrode implantation

The animal was administered a subcutaneous injection of atropine sulfate (0.01 mg/kg b.w.) before surgery and then was anesthetized with an initial intraperitoneal (i.p.) injection of sodium pentobarbital (40–50 mg/kg b.w.). After anesthesia, the animal was fixed on the stereotaxic apparatus (RWD, Shenzhen, China). The tetrode array was then implanted in the left mPFC (AP 2.5–4.5 mm, ML 0.3–0.8 mm, 2.0–3.5 mm ventral to the brain surface) by slowly advancing a micromanipulator (RWD, Shenzhen, China). Neuronal signals were monitored throughout implantation to ensure appropriate placement. Tissue gel (3 M, Maplewood, MN, US) was used to seal the craniotomy. The tetrode array was then secured to the skull with stainless steel screws and dental acrylic. After surgery, animals were given a 4-day course of antibiotics (Baytril, 5 mg/Kg b.w., Bayer, Whippany, NJ, US). They had a recovery period of at least 7 days (usually 9–12 days with free access to food and water).

### Neural recordings

When recovered from the surgery, animals resumed performing the behavioral task in the same training chamber but now situated inside a larger acoustically and electrically shielded room (size 2.5 × 2 × 2.5 m, length × width × height). Recording sessions began after the animal's behavioral performance recovered to the level attained before surgery (typically 2–3 days). Wideband neural signals (250–6000 Hz) were recorded using a head-stage amplifier (RHD2132, Intantech, CA, USA). Amplified (20×) and digitized (at 20 kHz) neural signals were combined with trace signals representing both the stimuli and session performance information and sent to a USB interface board (RHD2000 Intan technology, CA, USA), and then to a PC for on-line observation and data storage.

### Histology

After the last data recording session, the final tip position of the recording electrode was marked with a small DC lesion (-30 μA for 15 s). Afterwards, rats were deeply anesthetized with sodium pentobarbital (100 mg/kg) and perfused transcardially with saline for several minutes, followed immediately by phosphate-buffered saline (PBS) with 4% paraformaldehyde (PFA). Their brains were carefully removed and stored in the 4% PFA solution overnight. After cryoprotection in PBS with 20% sucrose solution for at least three days, the fixed brain tissue was sectioned in the coronal plane on a freezing microtome (Leica, Wetzlar, Germany) at a slice thickness of 50 μm and counterstained with methyl violet to aid lesion site verification to be in the Prelimbic area of mPFC [74, 75].

### Data analysis

Reaction time was defined as the time between the onset of a stimulus and the moment when the animal withdrew its nose from the infrared beam monitoring point in the central port. The average reaction time for each cue condition was calculated as the median over the number of trials given. The correct performance rate was defined by:

Correct performance rate (%) = 100*hit trials/ total number of trials.

Raw neural signals were recorded and stored for offline analysis. Spike sorting was later performed using Spike 2 software (CED version 8, Cambridge, UK). Recorded raw neural signals were band-pass filtered in 300–6000 Hz to remove field potentials. A threshold criterion of no less than threefold standard deviations (SD) above background noise were used for identifying spike peaks. The detected spike waveforms were then clustered by principal component analysis and a template-matching algorithm. Waveforms with inter-spike intervals of < 2.0 ms were excluded. Relative spike timing data for a single unit were then obtained for different trials of different cued conditions and used to construct both raster plots and prestimulus time histograms (PSTHs) using custom Matlab scripts. Only neurons for which the overall meaning firing rate within the session was at least 2 Hz were included for analysis. As generally observed, behavioral and neuronal results were similar across all relevant animals for a particular testing paradigm. Thus, the data across sessions were combined to study population effects.

To render PSTHs, all spike trains were first binned at 10 ms and convolved with a smoothing Gaussian Kernel (δ = 100 ms) to minimize the impact of random spike-time jitter at the borders between bins. The mean spontaneous firing rate was calculated from a 500-ms window immediately preceding stimulus onset. Decision-making-related neural activity was quantified as mean firing rates in the 500-ms after cue onset after subtracting the mean spontaneous firing rate.

We quantified the choice selectivity between two different cue conditions used during a task (for example, low tone trials vs. high tone trials) by using a receiver operating characteristic (ROC) based analysis [76]. Firstly, we set 12 threshold levels of activity covering the range of firing rates obtained in cue_A and cue_B trials. Following that, a ROC curve is generated, for each threshold criterion, by plotting the proportion of cue_A trials on which the response exceeded criterion against the proportion of cue_B trials on which the response exceeded criterion. The value of choice selectivity is defined as 2*((area under the ROC curve)–0.5). Therefore, a value of 0 indicates no difference in the distribution of responses between cue_A and cue_B. A value of 1/-1 represents the highest selectivity, that is, responses triggered by cue_A were always higher or lower than those evoked by cue_B.

To test the significance of each choice selectivity value, we ran a permutation test. This was accomplished by randomly distributing all trials from a neuron into two groups, independent of the actual cue conditions. These groups were nominally called cue_A trials and cue_B trials and contained the same number of trials as the experimentally obtained groups. The choice selectivity value was then calculated from the redistributed data, and the procedure was repeated 5000 times, thereby giving a distribution of values from which to calculate the probability of the result we obtained. When our actual value was found in the top 5%, it was defined as significant (i.e., *p* < 0.05).

To quantify the difference between responses in visual-auditory (multisensory) and auditory trials, we calculate the index of cross-modal interaction (MI) using the following function: MI = (VA-A)/(VA + A), where VA and A represent firing rates in multisensory and auditory trials, respectively. MI has a range of -1 to 1, with more positive values indicating the response in multisensory trials was much stronger and more negative values meaning the response in auditory trials was more robust.

### Statistical analysis

All statistical analyses were conducted in Matlab 2015b with statistical significance assigned for findings attaining a p-value of < 0.05. All behavioral data (for example, mean reaction time differences between auditory and multisensory trials) were compared using the paired t-test. We performed the Chi-square test to analyze the difference in proportions of neurons (recorded in different Tasks) showing choice selectivity. To compare MIs between different cue conditions within the same group of neurons, we performed a paired t-test or Mann–Whitney Rank Sum Test where appropriate. Unless stated otherwise, all data group results are presented as mean ± SD.

## Data Availability

The datasets generated and analyzed during the current study are available from the corresponding author on reasonable request.

## Abbreviations

CMI: Cross-modal interaction
IL: Infralimbic cortex
MI: Index of cross-modal interaction
mPFC: Medial prefrontal cortex
PBS: Phosphate-buffered saline
PFA: Paraformaldehyde
PrL: Prelimbic cortex
PSTH: Prestimulus time histogram
ROC: Receiver operating characteristic
SD: Standard deviations
SPL: Sound pressure level
